# The time course of ineffective sham‐blinding during low‐intensity (1 mA) transcranial direct current stimulation

**DOI:** 10.1111/ejn.14497

**Published:** 2019-07-08

**Authors:** Robert Greinacher, Larissa Buhôt, Lisa Möller, Gemma Learmonth

**Affiliations:** ^1^ School of Psychology University of Glasgow Glasgow UK; ^2^ Quality and Usability Lab Technische Universität Berlin Berlin Germany; ^3^ Department of Neurology University of Lübeck Lübeck Germany; ^4^ Centre for Cognitive Neuroimaging Institute of Neuroscience and Psychology University of Glasgow Glasgow UK

**Keywords:** placebo, primary motor cortex, reaction time, sham, tDCS

## Abstract

Studies using transcranial direct current stimulation (tDCS) typically compare an active protocol relative to a shorter sham (placebo) protocol. Both protocols are presumed to be perceptually identical on the scalp, and thus represent an effective method of delivering double‐blinded experimental designs. However, participants often show above‐chance accuracy when asked which condition involved active/sham retrospectively. We assessed the time course of sham‐blinding during active and sham tDCS. We predicted that participants would be aware that the current is switched on for longer in the active versus sham protocol. Thirty‐two adults were tested in a preregistered, double‐blinded, within‐subjects design. A forced‐choice reaction time task was undertaken before, during and after active (10 min 1 mA) and sham (20 s 1 mA) tDCS. The anode was placed over the left primary motor cortex (C3) to target the right hand, and the cathode on the right forehead. Two probe questions were asked every 30 s: “Is the stimulation on?” and “How sure are you?”. Distinct periods of non‐overlapping confidence intervals were identified between conditions, totalling 5 min (57.1% of the total difference in stimulation time). These began immediately after sham ramp‐down and lasted until the active protocol had ended. We therefore show a failure of placebo control during 1 mA tDCS. These results highlight the need to develop more effective methods of sham‐blinding during transcranial electrical stimulation protocols, even when delivered at low‐intensity current strengths.

## Graphical abstract

Low‐intensity (1 mA) anodal transcranial direct current stimulation was applied to the primary motor cortex for 10 min and compared to a 20 s sham protocol in the same individuals. Probe questions asked at regular intervals during the two protocols identified that participants were confident that the stimulation was active for a longer period during the 10 min stimulation compared to the 20 s sham protocol. We show here a failure of placebo control during the course of low‐intensity tDCS.

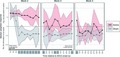

**Abbreviations**
CI: confidence interval
DC: direct current
EEG: electroencephalography
RT: reaction time
SD: standard deviation
tDCS: transcranial direct current stimulation
TOST: two one‐sided *t*‐tests

## 1 INTRODUCTION

Transcranial direct current stimulation (tDCS) is a popular, non‐invasive method of modulating cortical excitability. One of the main advantages of tDCS over other forms of neuromodulation, such as transcranial magnetic stimulation, is the ability to administer a placebo control protocol (sham tDCS) that is assumed to be perceptually indistinguishable from active stimulation on the scalp. In a typical sham protocol, the current is ramped‐up gradually, then delivered for a short period at the same intensity as the active comparator (≤2 mins at 1–2 mA), followed by a fade‐out phase. This brief stimulation period seems not to induce any substantial neuromodulatory effects (Nitsche et al., 2008) but enables the initial cutaneous sensations associated with stimulation, including tingling, itching, burning and headache (Brunoni et al., 2011; Gandiga, Hummel, & Cohen, 2006; Poreisz, Boros, Antal, & Paulus, 2007), to be presented in both the active and sham protocols.

Studies involving sham protocols therefore rely on participants being unable to differentiate the sensations experienced during this brief sham period from a prolonged period of active stimulation (10–20 mins). This is of vital importance to tDCS research, because a failure of sham‐blinding could potentially encourage participants to modify their behaviour, even subconsciously, in response to knowing whether or not they are receiving an active protocol. This may be particularly compromising to a study where the hypotheses and outcome measures are communicated to participants beforehand, for example via the study information sheets (Rabipour, Wu, Davidson, & Iacoboni, 2018). The effectiveness of sham‐blinding has been quantified in two broad ways in the prior literature: (a) by comparing the reported frequency and severity of cutaneous sensations during different protocols and (b) by asking participants to guess whether they had received active or sham tDCS (in the case of between‐groups designs), or which of multiple sessions had involved active or sham (in within‐group designs).

There is mixed evidence as to whether sham‐blinding can indeed be successfully achieved during the application of standard tDCS parameters. Broadly, low‐intensity (1–1.5 mA) protocols can be blinded more effectively than high‐intensity (2 mA) tDCS (O'Connell et al., 2012). However, some studies find better‐than‐chance rates of guessing which condition had involved active/sham at both 2 mA (O'Connell et al., 2012; Wallace, Cooper, Paulmann, Fitzgerald, & Russo, 2016) and 1–1.5 mA (Benwell, Learmonth, Miniussi, Harvey, & Thut, 2015; Goldman et al., 2011; Learmonth et al., 2017; O'Connell et al., 2012; Turi et al., 2019) current strengths, but others only around chance‐level during high‐ (Gandiga et al., 2006; Nitsche et al., 2003; Palm et al., 2013; Russo, Wallace, Fitzgerald, & Cooper, 2013; Tang, Hammond, & Badcock, 2016) and low‐ (Ambrus et al., 2012; Gandiga et al., 2006; Poreisz et al., 2007) intensity stimulation. However, these questions are typically probed on a retrospective basis, at the end of each session or experiment, and are therefore heavily reliant on the participants’ recollection of each event.

Indeed, there is scant evidence as to whether participants are able to dissociate active tDCS from sham during the course of an experiment. To assess this, Ambrus et al. (2012) probed the time course of cutaneous sensations at regular intervals during 10 min of 1 mA anodal stimulation, 10 min of 1 mA cathodal, and a 30 s sham protocol. Participants were asked to rate the perceived strength of sensations felt on the scalp at seven probe points, every 1.75 min after tDCS onset (scale = “no sensation” to “extreme discomfort”). There was no difference in perceived sensation strength between the three conditions at the first time point. The perceived strength then reduced significantly in the sham condition from 2.25 min onwards, from 4 min in the anodal, and from 5.75 min in the cathodal condition, but there were no statistical differences between the three conditions. Specifically, Ambrus et al. (2012) argued that active and sham protocols are perceptually indistinct due to the persistence of sensations in the sham condition that endure after stimulation has ended. Although this study provides important information regarding the time course of cutaneous sensations during tDCS, we argue that the question posed does not adequately probe whether participants can tell that the stimulation is active at present. It is feasible that active and sham protocols may be similar in their subjective (dis)comfort ratings, yet participants still able to identify when the stimulation is active due to additional factors that are not probed by this question.

In this study, we aimed to assess the time course of sham‐blinding by probing at 30 s intervals whether participants can identify that 1 mA tDCS is active or inactive, and the confidence of their decision. We chose to extend a well‐powered, preregistered reaction time experiment of Minarik et al. (2016), in which a small improvement in reaction time was elicited during 1 mA of anodal tDCS to the primary motor cortex, relative to a cathodal protocol. Here, we incorporated a 20 s sham protocol instead of the cathodal condition. We hypothesised that (a) participants will be able to identify that a low‐intensity current is switched on for a longer duration during the 10 min active protocol, compared to the 20 s sham, and (b) active anodal tDCS will reduce reaction times more effectively than sham.

## 2 MATERIAL AND METHODS

### 2.1 Preregistration

The study protocol was preregistered at https://osf.io/2zwhg/. The stimulus materials and full datasets are also available here.

### 2.2 Participants

An a priori sample size calculation, based on the effect size of *d* = 0.45 observed in Minarik et al. (2016), identified that 32 participants were required for a one‐tailed, repeated‐measures *t*‐test, where power = 0.8 and *α* = 0.05 (Faul, Erdfelder, Buchner, & Lang, 2009). Participants had a mean age of 24.5 years, range = 19–38, and 27 were female. All were right handed, reported normal or corrected‐to‐normal vision, had no tDCS contraindications (as per Rossi, Hallett, Rossini, & Pascual‐Leone, 2009) and had not received any form of electrical stimulation before. The study was approved by the University of Glasgow College of Science and Engineering ethics committee and performed in accordance with the Declaration of Helsinki. Written, informed consent was obtained from each participant.

### 2.3 tDCS

A direct current was applied using a battery‐driven constant current stimulator (NeuroConn GmbH, Germany). Two protocols were administered in a double‐blinded, counterbalanced, within‐subjects design with ≥24 hr between sessions: (a) ACTIVE ANODAL: 1 mA tDCS for 10 min, and (b) SHAM: 1 mA tDCS for 20 s, both with additional 30 s ramp‐up and 30 s ramp‐down periods. In both protocols, the anode was placed vertically over the left primary motor cortex (C3 of the 10–20 International EEG system) and the cathode horizontally over the right forehead. Both carbon rubber electrodes measured 5 × 7 cm and were encased in 0.9% NaCl saline‐soaked sponges, held in place using rubber bands. The “study mode” of the NeuroConn stimulator was used to double‐blind the protocols using five‐digit codes provided in the NeuroConn DC Stimulator user manual. The list of codes was generated by author GL and the list of conditions that corresponded to each code was stored on a PC that was not accessible to the experimenters (authors RG, LB and LM). Half of the codes initiated a preprogrammed active stimulation protocol and the other half the sham protocol. During the sham protocol, the device delivered a weak probe current of 110 μA every 550 ms to test the electrode impedance, which was relayed to the screen of the device. Thus, in both conditions, the screen displayed continuously updating impedance values, and a stimulation countdown time of 10 min, ensuring that the experimenters were blind to the protocol being delivered. Electrode impedance was 6.2 kΩ on average at the start of stimulation (range = 4.1–10.7 kΩ). Participants were given no information regarding the stimulation parameters (see information sheets provided at https://osf.io/2zwhg/), and experimenters were specifically instructed not to answer direct questions from the participants about the study until all datasets had been collected.

### 2.4 Reaction time task and sham‐blinding probes

**Figure 1:**
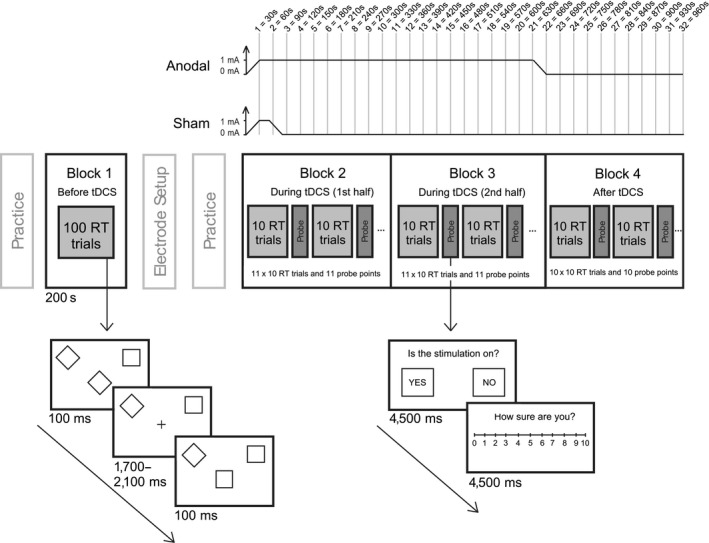
Illustration of the experimental design. Block 1 was performed as a baseline measure before tDCS onset. The stimulation ramp‐up in both conditions began at the start of Block 2 and the active ramp‐down finished at the end of Block 3. In Blocks 2–4, sham‐blinding probe questions were inserted at 30 s intervals to assess (1) whether participants could identify when the stimulation was switched on and (2) the confidence of their decision

The reaction time task was adapted from Minarik et al. (2016) and was performed on a Dell Precision T3400 PC and 19.5′′ Sun Microsystems CRT monitor with 1,280 × 1,024 pixel resolution and 100 Hz refresh rate. The task in Block 1 (baseline) was identical to the procedure of Minarik et al. (2016): either a square or a diamond appeared in the centre of the screen for 100 ms, followed by a fixation cross of variable duration between 1,700–2,100 ms (Figure 1). Participants were instructed to press the left mouse button if they saw a diamond and the right mouse button for a square, as quickly and accurately as possible. The original stimuli of Minarik et al. were amended by affixing a diamond to the upper left corner and a square to the upper right throughout the experiment, as piloting highlighted that some participants erroneously switched responses mid‐experiment. In Block 1, a total of 100 trials lasting 200 s were presented. In Blocks 2–4, we inserted two probe questions into the reaction time task at 30 s intervals, after every 10 trials, with each probe question presented on the screen for 4,500 ms. Question 1 involved a binary yes/no choice (“Is the stimulation on?”), and question 2 assessed the confidence of their decision (“How sure are you?”) via a visual analogue scale where 0 = very unsure and 10 = very sure. Questions were answered using a mouse click. The task was initiated in Block 2 at the same time as the 30 s tDCS ramp‐up began, with the first sham‐blinding probe point occurring 30 s after tDCS onset (i.e., at the point at which the stimulation reached 1 mA in both the active and sham protocols). The task was then performed for 16 min continuously: during the 30 s ramp‐up, the 10 min of online tDCS (active), the 30 s ramp‐down, then for 5 min offline after the active tDCS had ended. There were 220 trials during stimulation (Blocks 2 & 3) and a further 100 trials after stimulation had ended, providing a total of 32 probe points to assess the time course of sham‐blinding.

### 2.5 Procedure

Participants completed a short practice block (30 trials) of the reaction time task, followed by the baseline Block 1. The electrodes were then applied to the scalp. The tDCS device was programmed using a preallocated five‐digit code and the resistance was checked and lowered if necessary. A short practice of the reaction time task, including the sham‐blinding probes, was performed. Block 2 of the behavioural task was initiated at the same time that the stimulator began its ramp‐up to 1 mA. Electrode resistance was recorded when the tDCS device reached 1 mA. Blocks 2–4 were then completed in a single, continuous 16 min period. The stimulation ramp‐down coincided with the end of Block 3 in the active condition. Block 4 was performed after active tDCS. After the electrodes were removed, participants rated whether they had experienced headache, tingling, itching, burning or pain during the session (1 = Not at all, 5 = Very strongly). At the end of the second session, they were informed about the two protocols and asked to guess which of the two sessions had involved sham, and the confidence of their guess (scale = 1–10). In contrast to the between‐groups design of Minarik et al. (2016), here participants attended on two different days, a minimum of 24 hr apart, in a within‐subjects design. Finally, after all 32 participants had been tested, the researchers were un‐blinded to the stimulation sessions and the data sorted into active and sham sessions.

## 3 RESULTS

### 3.1 Effectiveness of sham‐blinding

**Figure 2:**
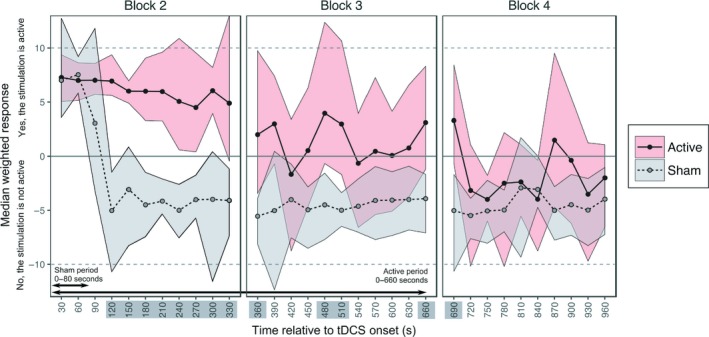
Sham‐blinding results. The median weighted responses are shown for active and sham, with bootstrapped 95% confidence intervals. Time points with distinct (i.e., non‐overlapping) ratings for active and sham are highlighted. [Colour figure can be viewed at http://wileyonlinelibrary.com]

We had preregistered a plan to fit binomial logistic curves for each individual and calculate the time point within the experiment at which a 50/50 yes/no guess rate was reached. Unfortunately, the data were found to be unconducive to this analysis due to participants switching between yes and no responses more frequently than anticipated. It should therefore be noted that these results are derived from a post hoc alteration to the analysis plan. The outcome measures derived from the two probe questions (Is the stimulation on? and How sure are you?) were combined to create a weighted score. A “yes” response to the first question was assigned a value of +1 and “no” a value of −1. This was then multiplied by the confidence rating (0–10), so that a value of +10 indicated high confidence that the stimulation was switched on, and −10 high confidence that it was switched off. We then bootstrapped 95% confidence intervals for each of the 32 probe points, separately for the active and sham conditions, using 5,000 permutations of the data (Figure 2).

We identified overlapping confidence intervals between the active and sham protocols throughout the first 90 s of stimulation, where participants were confident that the tDCS was switched on in both conditions. This period included the 30 s ramp‐up in both protocols, followed by the 20 s 1 mA sham tDCS and the 30 s sham ramp‐down. In the active condition, confidence intervals remained above zero until 5 min (300 s) post‐tDCS onset. Group‐level responses became more varied from this point onwards, with wider confidence intervals that overlapped zero, although the median value generally remained above zero until the active stimulation had ended. Conversely, in the sham condition, participants were confident that no stimulation was being delivered from 2 min (120 s) post‐tDCS onset and this lasted broadly until the end of the experiment. Comparing active and sham directly, the confidence intervals were distinct for a total of 5 min and this represented 51.7% of the total difference in stimulation time between conditions (see highlighted time points on the *x*‐axis of Figure 2). The first non‐overlapping period started at 120 s post‐tDCS onset and lasted until 360 s, a second period was identified between 480–510 s and a third between 660 and 690 s (the active protocol had fully ramped‐down by 660 s). Finally, the weighted scores were no different between active and sham during Block 4, when the stimulation had ceased in both protocols, from 12 min (720 s) until the end of the experiment.

### 3.2 Reaction times

**Figure 3:**
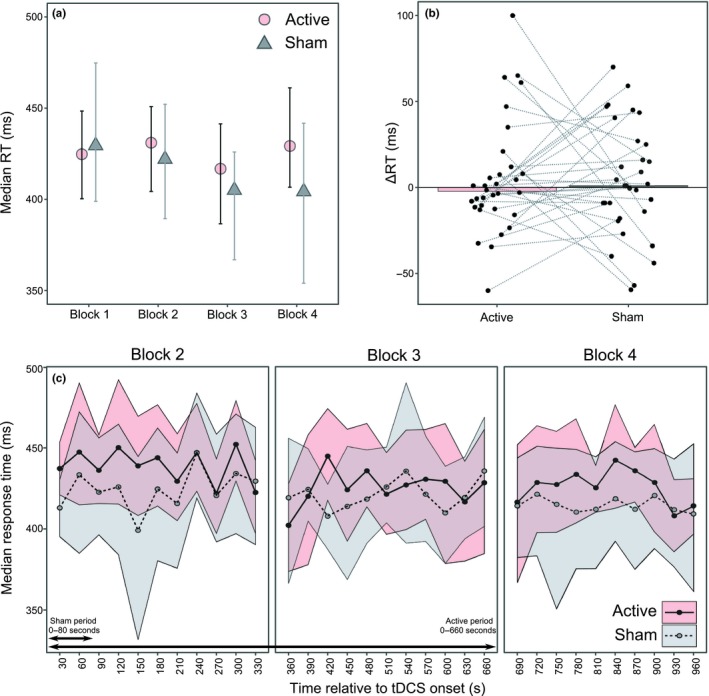
Reaction time results. (a) Median group‐level RTs per block with bootstrapped 95% CIs. (b) Median change in RT from baseline to Block 3 with individual participant medians shown. (c) Median RTs for each sub‐block of 10 trials with bootstrapped 95% CIs. [Colour figure can be viewed at http://wileyonlinelibrary.com]

Participants were highly accurate in the behavioural task throughout the experiment (mean accuracy = 95.6%, bootstrapped 95% CI = [95.08, 96.11%]). As per the preregistered analysis plan, the median response time for correct trials was calculated for each participant per block (Figure 3a). Reaction time at baseline (pre‐tDCS), averaged across anodal and sham conditions was 433.57 ms, *SD* = 58.68, and during Block 3 (the second half of stimulation) was 429.25 ms, *SD* = 57.78. These RTs were no different to those obtained in Minarik et al. (2016) (at baseline *t*(47.72) = 0.76, *p* = 0.45, mean = 442.36 ms, *SD* = 45.63 and during stimulation *t*(49.14) = 0.34, *p* = 0.74, mean = 433.13 ms, *SD *= 46.77, data available from https://osf.io/xnyar/?view_only=2743a0c4600943c998c2c37fbfb25846). A TOST procedure indicated that baseline reaction times were equivalent in both conditions (i.e., the observed effect size of *d*_*z*_ = −0.02 was within the equivalent bounds of a moderate effect size of *d*_*z*_ = −0.4 and 0.4; *t*(31) = 2.15, *p* = 0.02 (Lakens, 2017)). The baseline RTs (Block 1) were then subtracted from each subsequent block to create a ΔRT value for each of Blocks 2–4 (Figure 3b). A series of three one‐tailed, repeated‐measures *t*‐tests were performed to compare the ΔRT in the active and the sham protocols, separately per block. There were no differences in RT shifts from baseline between the active and sham conditions in any of the 3 blocks (ΔRT Block 2: *t*(31) = 0.26, *p* = 0.4, *d* = 0.042; Block 3: *t*(31) = −0.16, *p* = 0.44, *d* = 0.028; Block 4: *t*(31) = −0.29, *p* = 0.39, *d* = 0.047). As an exploratory follow‐up, we assessed whether there were any transient differences between anodal and sham tDCS that might have been obscured by collapsing the reaction time data into 5‐min blocks. The median reaction times were bootstrapped for each sub‐block of 10 trials in Blocks 2–4, but no non‐overlapping periods were observed between conditions (Figure 3c).

### 3.3 Side effects and sham‐blinding questionnaires

Wilcoxon signed‐rank tests were performed to compare the ratings obtained in the active and sham conditions. Five tests were performed in total, one for each of the five sensory side effects (tingling, itching, headache, burning and pain). These identified a significant difference in the retrospective rating of itchiness between the active and sham conditions (*Z* = −3.13, *p* = 0.002). There were no differences for ratings of headache (*Z *= −0.18, *p* = 0.86), tingling (*Z* = −1.29, *p* = 0.197), burning (*Z* = −1.25, *p* = 0.21) or pain (*Z* = −0.979, *p* = 0.33). Participants were above chance (25/32 = 78.1% correct) in their ability to guess which of the two sessions had involved sham at the end of the experiment.

## 4 DISCUSSION

We present here two main findings. Firstly, participants were confident that active stimulation was being delivered for a longer duration during 10 mins of active anodal tDCS compared to a 20 s sham protocol. Secondly, 10 min of 1 mA anodal tDCS to the primary motor cortex failed to reduce reaction times relative to sham. These results add to previously reported null findings relating to stimulation of the primary motor cortex (Apšvalka, Ramsey, & Cross, 2018; Conley, Fulham, Marquez, Parsons, & Karayanidis, 2016; Conley et al., 2015; Horvath, Vogrin, Carter, Cook, & Forte, 2016; Turkakin et al., 2018). Since Minarik et al. (2016) did not include a sham protocol, it was not previously possible to determine whether the anodal tDCS resulted in improved reaction times, or whether the cathodal protocol had actively inhibited motor learning throughout the task. We found that our sham protocol elicited a similar change in reaction time to their cathodal condition, but here we failed to find a facilitation of reaction times in response to anodal tDCS. However, we should emphasise that we modified both the task and experimental design in the present study and as such, we would not expect to observe the same magnitude of difference in ΔRT values when comparing anodal vs. sham here as when comparing anodal vs. cathodal protocols. Nevertheless, these results do indicate that the small improvement in reaction time afforded by anodal tDCS is likely to be an unstable finding and can be eliminated simply by changing the experimental design from between‐groups to a within‐subjects approach. We have previously documented this instability in a failed replication of our own work (see Benwell et al., 2015; Learmonth et al., 2017). A second difference between the two studies is that after baseline testing, Minarik et al. (2016) delivered 4 min of tDCS offline, prior to 4 min online tDCS, during which reaction times were re‐assessed. Our active protocol was delivered entirely online, and there is current debate regarding whether anodal stimulation is more effective in modulating motor cortex excitability when applied before, rather than during, behavioural tasks (Cabral et al., 2015).

Our results also highlight a failure of sham‐blinding during low‐intensity tDCS, adding to previous similar results at a higher current (O'Connell et al., 2012) and adding important information regarding the time course of failed sham‐blinding. Specifically, we show that the cessation of stimulation in the sham protocol resulted in an immediate and sharp increase in the number of participants who were confident that the stimulator had been deactivated, which lasted until the end of the experiment. It is important to note that participants were given no prior information about the two stimulation conditions and the experimenters were also blinded to the counterbalancing across conditions. These results contradict prior claims that active and sham protocols are perceptually indistinct due to a persistence of sensations after the ramp‐down has ended in sham protocols (Ambrus et al., 2012). In the active protocol, we found a maintained confidence that the stimulation was still switched on until 5 mins into the stimulation period, and periods of difference relative to sham that lasted broadly until the stimulation had ended in the active condition. We did, however, observe a gradual reduction in group‐level confidence over the course of the 10 min anodal stimulation, with an increased variability of responses within the group, supporting the general concept of scalp desensitisation during sustained active tDCS. However, we argue that placebo controls must remain consistently blinded over the whole time course of application to be deemed effective, particularly if participants are made aware of protocol differences beforehand (Davis, Gold, Pascual‐Leone, & Bracewell, 2013; Rabipour et al., 2018).

Our findings provide new and important information regarding the time course of sham‐blinding that could only be revealed by probing this issue throughout the experiment, rather than retrospectively. Studies that probe whether sham‐blinding was achieved on a retrospective basis could potentially introduce a number of confounding variables that may influence the accuracy of these reports. Firstly, participants may have a poor recollection of what they experienced during each session, particularly if there were multiple sessions with long periods between testing days. Secondly, the probe questions may not allow for nuanced responses relating to the certainty of these decisions and what factors might have led to them, nor the duration of any side effects experienced. For instance, we often anecdotally find that participants report the tightness of the rubber bands to be the most uncomfortable aspect of receiving tDCS. It is possible that participants could assign a disproportionate weighting to such factors in their reports, which are unrelated to stimulation *per se*, and would likely be identical across tDCS conditions. Such factors could account for the mixed reports in the prior literature (Benwell et al., 2015; Gandiga et al., 2006; Goldman et al., 2011; Learmonth et al., 2017; Nitsche et al., 2003; O'Connell et al., 2012; Palm et al., 2013; Russo et al., 2013; Tang et al., 2016; Wallace et al., 2016). In going some way to address this issue, Ambrus et al. (2012) found no difference in the time course of *discomfort* ratings between active and sham tDCS, which was interpreted as successful sham‐blinding. However, here we find prolonged differences when participants are asked specifically whether or not they believed the stimulator to be active.

It is important, then, to consider possible methods of delivering more effective sham‐blinding during electrical stimulation studies. Topical anaesthetics can reduce or eliminate skin sensations associated with tDCS (Guleyupoglu, Febles, Minhas, Hahn, & Bikson, 2014; McFadden, Borckardt, George, & Beam, 2011), although might mask more serious side effects such as skin burning (Palm et al., 2008). However, this appears to be low risk using standard stimulation parameters (Loo et al., 2011). A second option is to compare anodal stimulation against a cathodal paradigm, without including a sham, which should give rise to observable differences in outcome measures. This approach is often precluded, e.g., in certain clinical groups such as stroke, where it would be counterintuitive to deliver a protocol which further impedes behaviour. A third option is to compare the outcomes of two different electrode montages, one targeting the cortical area of interest and the second a location uninvolved in that behaviour (“off‐target active stimulation” (Davis et al., 2013)). It is also possible to reduce priming effects caused by participant expectations by using de facto masking, where participants are erroneously informed that the stimulation is active in all sessions (O'Connell et al., 2012). However, it is unclear whether this form of masking could successfully counteract the sensory differences observed between protocols as described here. In fact, our participants were informed that “weak electric current will be applied over your scalp for several minutes” but they remained able to tell when the stimulation ended during the sham protocol. We suggest that the appropriate control should be carefully tailored based on the specific aims and participant population of each individual study. We also recommend that researchers ensure that the aims, hypotheses and study design are concealed in any information sheets provided to participants to minimise demand characteristics associated with identifying the active stimulation condition.

It could be argued that by directly asking participants whether the stimulation is active, we may have drawn attention to this issue more than in a typical experiment. Given that the reaction times here were no different to those obtained in Minarik et al. (2016), either at baseline or during stimulation, we do not believe participants to have been distracted from the task by these probe questions, although we now aim to address this directly in follow‐up studies. We also reason that the effectiveness of placebo control should not be reliant upon participants being able to distract themselves from thinking about the conditions that are being administered. Secondly, it is empirically unknown what participants *do* think about during stimulation experiments, and this factor cannot be easily tested nor controlled. Although participants are instructed to focus on a task during online tDCS delivery, they may be simultaneously thinking about the sensations on their scalp. We would then also expect to find higher rates of failed sham‐blinding in offline experiments, when participants are instructed to rest during stimulation with no task to distract from these sensations. A second consideration is that our participants experienced both stimulation conditions and were able to compare sensations across days. However, within‐subjects and crossover designs are common in electrical stimulation studies, particularly in clinical trial designs, and thus achieving sham‐blinding in these designs remains a pertinent issue. Further, a systematic review of the current literature should be undertaken to quantify the influence of other experimental factors, such as the information provided to participants prior to the study (Rabipour et al., 2018), their expectations (Ambrus et al., 2012) and whether the experimenters were blind to the protocols being administered. We also recommend that further, basic methodological studies be conducted to develop improved sham protocols that can be adequately blinded from active electrical stimulation.

## 5 CONCLUSIONS

We conclude that the standard method of sham‐blinding reported here may be ineffective in healthy young adults, and could allow participant expectations to influence the study outcomes. These results highlight a need to develop more effective methods of delivering blinded placebo control protocols during electrical stimulation experiments, and specific assessment of this issue should now be undertaken within other populations, such as clinical groups and older adults.

## CONFLICT OF INTEREST

The authors declare no competing interests.

## AUTHOR CONTRIBUTIONS

GL conceived and designed the experiment. RG, LB, LM performed the experiments. GL, RG, LB analysed the data. GL, RG, LB, LM prepared the manuscript.

## Data Availability

The preregistered protocol, stimulus materials and full datasets are available at https://osf.io/2zwhg/.
